# Management of Cancer-Associated Venous Thrombosis: A Nationwide Survey among Danish Oncologists

**DOI:** 10.1055/s-0041-1729754

**Published:** 2021-06-16

**Authors:** Anette Arbjerg Højen, Thure Filskov Overvad, Mads Nybo, Thomas Kümler, Morten Schnack Rasmussen, Thomas Decker Christensen, Torben Bjerregaard Larsen

**Affiliations:** 1 Department of Cardiology, Aalborg University Hospital, Aalborg, Denmark; 2 Aalborg Thrombosis Research Unit, Department of Clinical Medicine, Faculty of Health, Aalborg University, Aalborg, Denmark; 3 Department of Oncology, Aalborg University Hospital, Aalborg, Denmark; 4 Department of Clinical Biochemistry and Pharmacology, Odense University Hospital, Odense, Denmark; 5 Department of Cardiology, Herlev- Gentofte Hospital, Copenhagen, Denmark; 6 Department of Surgical Gastroenterology, Bispebjerg Hospital, University of Copenhagen, Denmark; 7 Department of Cardiothoracic and Vascular Surgery and Department of Clinical Medicine, Aarhus University Hospital, Aarhus, Denmark

**Keywords:** venous thromboembolism, cancer associated venous thrombosis, practice guideline, cancer, survey

## Abstract

**Background** Treatment patterns for cancer-associated venous thrombosis (CAT) has been shown to be nonconsistent with contemporary guideline recommendations, resulting in poor patient outcomes.

**Objectives** The study aimed to describe contemporary CAT management in Danish oncology departments and identify knowledge gaps and inconsistencies between guidelines and clinical practice.

**Patients and Methods** A survey questionnaire in Danish was developed based on contemporary national guidelines. Using an open recruitment strategy, invitations to participate in the electronic survey were sent to physicians employed at oncology departments in Denmark in winter of 2018/2019. The questionnaire was based on current national guidelines and included 10 items with multiple choices and a free-text option to specify or comment. The questionnaire was pilot-tested by a junior and senior oncologist.

**Results** A total of 142 physicians completed the survey, representing all Danish geographical regions and various seniority. The majority reported that CAT was treated and followed up in oncology departments. However, 36.6% of the physicians were unaware of the existence of designated cancer thrombosis guidelines. Risk of venous thrombosis was generally assessed without diagnostic scores. Almost all (98.6%) reported low-molecular-weight heparin to be first-line treatment for CAT. Treatment duration seemed wrongly influenced by subtype of venous thrombosis, and 44.5% responded that thromboprophylaxis among hospitalized patients was substantially underused.

**Conclusion** The variability in the daily clinical management of CAT demonstrated through this survey indicates a potential to increase awareness of available guidelines, standardized use of inpatient thromboprophylaxis, and organized treatment and follow-up in a multidisciplinary setting, which would potentially improve management of CAT in Denmark.

## Introduction

Venous thromboembolism is a frequent complication among cancer patients and is associated with increased morbidity, mortality, and psychological distress. 1 2 Management of cancer-associated venous thrombosis (CAT) poses a particular challenge because of a high risk of major bleeding and recurrent venous thrombosis compared with the noncancer population. 3 4

To aid in treatment decisions, numerous designated guidelines for CAT management have been developed. 5 6 7 In Denmark, the management of CAT has been supported by National Clinical Practice guidelines developed in collaboration between The Danish Society on Thrombosis and Hemostasis and The Danish Society for Clinical Oncology since 2009. 8 Nonetheless, several reports have revealed treatment patterns for CAT that were nonconsistent with contemporary guideline recommendations, including both underuse and inappropriate choice of anticoagulant agents. 9 10

However, how CAT is actually managed in daily clinical practice in Danish oncology departments has not been investigated.

The aim of this study was to describe contemporary CAT management in Danish oncology departments, hereby also potentially identifying knowledge gaps and inconsistencies between guidelines and clinical practice.

## Patients and Methods

### Study Design and Participants

This was a cross-sectional survey study describing the daily clinical management of CAT in the oncology departments in Denmark.

### Survey Design

A survey questionnaire in Danish was developed based on contemporary national guidelines by the Danish Society of Thrombosis and Hemostasis' working group on implementation of the Cancer and venous thromboembolism guideline. The survey was constructed and administered electronically using Research Electronic Data Capture (REDCap) electronic data capture tools hosted at (North Denmark region). 11 Survey questions was based on the recommendations in the 2017 National Guideline on Cancer and Venous Thromboembolism developed collaboratively by the Danish Society of Thrombosis and Hemostasis and The Danish Society for Clinical Oncology. 12 It included 18 questionnaire items covering 10 sections, evaluating diagnosis, anticoagulant therapy, treatment duration, bleeding risk, follow-up, thromboprophylaxis, recurrence, and treatment of deep vein thrombosis (DVT) and/or pulmonary embolism (PE) in the department as well as assessing demographic data including geographical region of employment in Denmark, seniority, oncology subspecialty. Questionnaire items, translated into English, are presented in Supplementary Table 1 (available in the online version). Response formats included yes/no, multiple choice, and open-ended text boxes. The participants were asked to answer based on the common practice in the department and, where appropriate, items provided a nonresponse (“do not know”) and/or “other” option. All questions had a free-text field for explanatory comments or specifications. Prior to submitting their responses, the physicians could change their answers using a back button to navigate backward to previous survey sections. The survey was pilot-tested by a junior and senior oncologist and refined according to their feedback. No incentives were given to participate. The survey was designed and reported according to the Checklist for Reporting Results of Internet E-surveys (CHERRIES). 13

**Table 1:** Demographic characteristics

Variable	% ( *n* )
Seniority	
Chief physician	36.6 (52)
Senior registrar	23.9 (34)
Registrar	25.4 (36)
Intern/resident	14.1 (20)
Region of Denmark	
Capital region of Denmark (1.76 million citizens)	28.9 (41)
Central Denmark region (1.28 million citizens)	21.8 (31)
North Denmark region (0.58 million citizens)	9.9 (14)
Region of Southern Denmark (1.2 million citizens)	32.4 (46)
Region Zealand (0.81 million citizens)	7.0 (10)

### Survey Dissemination and Data Collection

An open recruitment strategy was used. Invitations to participate were sent out via e-mail in late 2018 to physicians employed at oncology departments in Denmark. The Danish Society for Clinical Oncology and a contact person from each oncology department distributed the invitation letter by e-mail, including an embedded survey link and information about the purpose of the study, investigators, and length of the survey. Reminder e-mails were sent out in January 2019. The survey was open from November 30, 2018 to January 31, 2019.

### Statistical Analyses

Data were extracted from REDCap and analyzed via simple descriptive statistics using STATA/MP (v. 15.1). Only completed surveys were included in the analysis, and duplicates and invalid responses were removed. Fisher's exact test was used to compare nominal data on demographic differences between dropout participants and the larger sample. Open-ended responses were imported into NVivo, and content analyses was conducted by the first author. 14 The total number of responses varied between questions as adaptive questioning was used to reduce number and complexity of questions. The total number of responses for each survey question is included in Supplementary Table 1 (available in the online version).

## Results

### Respondent Characteristics

A total of 156 responded to the survey. Because of the open recruitment strategy, calculation of the exact response rate was not possible. 13 Based on the approximate number of registered oncologists (including residents) employed at the oncology departments in Denmark at the time, the estimated response rate was approximately 30%. 15 Of the 156 participants who commenced the survey, 7 participants did not answer any demographic questions and 14 participants completed the demographic questions, but did not otherwise complete the full survey. Fisher's exact test revealed no significant difference between these 14 participants and the participants who fulfilled the survey in terms of geographical region of employment in Denmark ( *p* = 0.925) or seniority ( *p* = 0.628) *.* In total, 142 participants completed the survey, resulting in a completion rate of 91%. More than half of the participants (55%, *n* = 78) specified or commended their answers in the open-ended textboxes, resulting in 201 free-text responses.

**Fig. 1:**
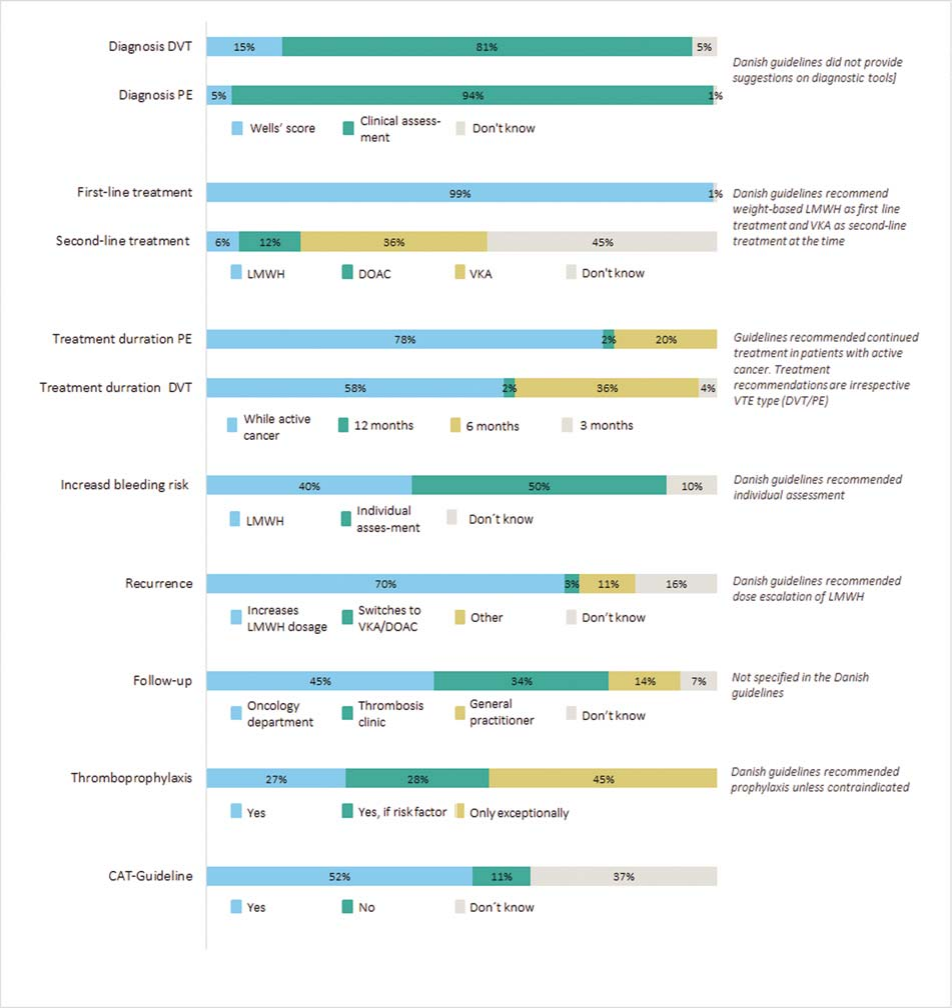
Management of cancer associated thrombosis in Danish oncology departments. CAT, cancer associated thrombosis; DOAC, direct oral anticoagulant; DVT, deep vein thrombosis; LMWH, low-molecular-weight heparin; PE, pulmonary embolism; VKA, vitamin K antagonist.

Demographic characteristics of the participants included in the analyses are presented in Table 1 . We received responses from physicians of various seniority employed in all geographical regions of Denmark. The participants covered a broad range of oncology subspecialities, the most frequently reported being lung cancer (19.0%, *n* = 27), gastrointestinal cancer (19.0%, *n* = 27), and breast cancer (18.3%, *n* = 26). Main results are presented in Fig. 1 , and detailed survey results are presented in Supplementary Table 1 (available in the online version).

### Cancer-Associated Venous Thrombosis Guidelines

Overall, 11.3% ( *n* = 16) of the participants reported that the department did not have clinical practice guidelines for management of CAT, and 36.6% ( *n* = 52) that they did not know. One of the participants who answered the department did have CAT guidelines elaborated that it was: “Not a general guideline, but several minor clinical practice guidelines” (chief physician).

### Treatment of Venous Thromboembolism in the Oncology Department

Almost all (98.6%, *n* = 140) participants reported that DVT was treated in the oncology department. With respect to PE, 62.7% ( *n* = 89) reported that they treat patients in the oncology department, and 35.9% ( *n* = 51) responded that they only treat patients with PE if they are hemodynamically stable. Patients not hemodynamically stable were reported to be most commonly treated at the cardiology department (86.3%, *n* = 44). One respondent specified that the treatment location for patients who were not hemodynamically stable depended on the capacity in the cardiology department: “If there is a shortage of beds, the patient is treated in the oncology department” (intern/resident).

### Diagnosis

Clinical assessment without the use of designated probability scores (e.g., Wells' scoring) was the most frequently used approach to assess the clinical probability of DVT (80.7%, *n* = 113) and PE (94.3%, *n* = 132). Few reported the Wells' score to be the most commonly used strategy (DVT 15.0%, *n* = 21; PE 5.0%, *n* = 7). Of note, several of the participants described a low threshold for diagnostic imaging in the free-text responses: “Ultrasound/computed tomography angiography/ventilation/perfusion scintigraphy on very low indication” (registrar). Further, one of the participants described a disagreement regarding the use of D-dimer testing in the diagnosis of CAT: “There is not—at least among the doctors who man our acute room—consensus, regarding whether we should use D-dimer test, etc. in the diagnostic process” (intern/resident).

### Anticoagulant Treatment

Almost all (98.6%, *n* = 138) reported low-molecular-weight heparin (LMWH) to be first-line treatment for CAT. However, the free-text responses indicated a growing expectation from patients to choose a direct oral anticoagulant (DOAC). “Our guideline refers to the DSTH CAT-report (…) although there is a growing expectation from the patients to choose a DOAC”(senior registrar). For second-line treatment, that is, cases where the patient either does not tolerate or accept first-line treatment, 36.4% ( *n* = 51) would choose a DOAC and 12.1% a vitamin K antagonist. Approximately, half responded that they did not know what second-line treatment would be. In the free-text responses, it was specified that in case of need for second-line agents, specialized advice would be sought. “If we cannot prescribe heparin, we seek advice from the thrombosis clinic” (senior registrar).

### Anticoagulant Treatment Duration

The majority (77.7%, *n* = 108) responded that patients with active cancer who present with PE are treated as long as they have active cancer, whereas 20.1% ( *n* = 28) responded they are treated for 6 months. For patients with new onset DVT, 58.3% ( *n* = 81) responded that patients are treated while cancer is ongoing, 36.0% ( *n* = 50) for 6 months, and 3.6% ( *n* = 5) that they are treated for 3 months. In the free-text responses, some of the participants specified that active cancer equaled active treatment. Thus, the patients would be treated with anticoagulants: “As long as the patient is receiving chemotherapy” (registrar). Others pointed out that active cancer may be interpreted in many ways and that it was often defined through discussion with local specialists: “Treatment duration is often discussed with the department's thrombosis specialist since the definition of active cancer can be interpreted in several ways” (senior registrar).

### Increased Bleeding Risk

In patients with increased bleeding risk, 40.3% ( *n* = 56) of the participants reported that the preferred treatment choice in the department was LMWH, and approximately half, 49.6% ( *n* = 69), reported that the choice of type and intensity of anticoagulant treatment is based on an individual assessment. Free-text comments elaborated that this is often based on advice from thrombosis specialists and always weighing in patients' preferences: “Always an individual assessment in collaboration with the patient” (senior registrar).

### Recurrence of Venous Thromboembolism

If patients experience recurrent VTE while treated with LMWH, 70.3% ( *n* = 97) of the participants responded that the treatment choice in the department was to increase the dosage of LMWH, while 10.9% ( *n* = 15) reported other treatment choices, including continuing current treatment, dividing current dose into two, and to seek specialist advice.

### Follow-Up

Follow-up care was reported to be primarily managed in the oncology department (44.5%, *n* = 61) and in specialized outpatient thrombosis clinics (34.3%, *n* = 47). However, free-text comments stated that follow-up care in the oncology department was regarded as passive follow-up during active oncology treatment: “No follow-up except what the patients already attends because of their cancer” (chief physician).

### Thromboprophylaxis for Immobilized Patients with Active Cancer

For immobilized patients with active cancer, almost half of the participants (44.6%) responded that thromboprophylaxis was only offered in exceptional cases. Thromboprophylaxis was recognized to have considerable improvement potential in the free-text responses: “We could definitely improve or focus on this” (chief physician). A recognition of lack of guideline adherence was also evident: “We could be better at starting it earlier. The clinical guideline is as such pretty clear in that respect” (senior registrar). Organizational structures were identified by one of the participants as a barrier for thromboprophylaxis. “It often fails because of the many different doctors at rounds, since nobody counts how many days the patient actually has been bedbound” (intern/resident).

## Discussion

This nationwide survey among Danish oncologists provided contemporary insights into practice patterns for CAT management. We found that CAT was often treated and followed up in oncology departments. Nonetheless, a substantial proportion of physicians were unaware of the existence of designated cancer thrombosis guidelines. Risk of venous thrombosis was generally assessed without diagnostic scores. Low-molecular-weight heparin was the most frequently used anticoagulant. Treatment duration seemed wrongly influenced by subtype of venous thrombosis, and thromboprophylaxis among hospitalized patients was substantially underused.

Our findings indicated that treatment and knowledge gaps about CAT remain despite the existence of both national and international guidelines. In line with our observations, a survey assessing attitudes and barriers in CAT treatment among cardiologists, hematologists, and oncologists, presented at the ISTH 2020 Congress, indicated a substantial lack of knowledge regarding the existence of designated cancer thrombosis guidelines, despite these being available for over a decade. 16 In addition to lack of knowledge, organizational constraints and strengths of habits play an important role in nonadherence to guidelines. 17 Indeed, our findings indicated that the treating physicians were aware that thromboprophylaxis was suboptimal and not distributed according to guidelines. They recognized there was room for improvement, and in line with previous observations, environmental-level extrinsic factors were identified as a barrier for guideline adherence. 18 Nevertheless, our findings still indicated intrinsic factor barriers concerning lack of familiarity with the CAT guidelines, as some of the free-text comments indicated thromboprophylaxis were to be initiated when the patient had already been bedbound for several days, and not when anticipating so. Indeed, attention on familiarity as well as awareness is essential when ensuring CAT guidelines adherence. Integration of guideline recommendations into the daily clinical workflow at the point of care, that is, using electronic reminders in the electronic medical records or an app supporting clinical decisions, will likely enhance awareness and familiarity with CAT guidelines. 17

Choice of anticoagulant agent for CAT in contemporary clinical practice remains nonadherent to guideline recommendations. 19 In this survey, almost all respondents suggested correctly that LMWH was the primary anticoagulant choice in the department. In addition, some reported DOACs as second line agents, which was not recommended in the Danish 2017 guidelines. Nevertheless, this likely reflects that some treating physicians were aware of the emerging role of DOACs in CAT management. 20 21 Nonetheless, a descriptive study using data from 2012 to 2017 from Denmark found that rivaroxaban was rarely used for CAT. 22 However, with the emerging role of DOACs in CAT management, and rapid uptake of recommendation on their use in guideline recommendations, this is likely to change in the years to come. 5 7 23 Current Danish cancer-thrombosis guidelines now recommend DOACs as first-line agents for selected cancer patients, but issues with more drug–drug interactions with DOACs versus LMWH does not necessarily make treatment decisions easier for the treating physician. 8 24 Although previous studies have shown that daily LMWH self-injections are well accepted by patients with CAT, our findings indicated the treating physicians experience a growing expectation from patients to choose a DOAC, supporting previous observations that patients with cancer generally prefer orally administered drugs if they are equally effective. 25 26 27 28 29 In a complex clinical situation as CAT, patients' needs and preferences are diverse, and incorporating the patients' attitude regarding the anticoagulant treatment is essential. 30 Concordant, many of the free-text responses indicated that treating physicians in this survey included patient preferences when weighing treatment decisions.

Decisions on treatment duration are traditionally challenging and also sparsely supported by randomized data. 31 Danish recommendations for duration of anticoagulant treatment for CAT are similar irrespective of the subtype of venous thrombosis. Nonetheless, the reported treatment duration differed for DVT and PE in the participant responses, where treating physicians were more likely to provide extended anticoagulation for patients with PE (77.7%) compared with patients with DVT (58.3%). Thus, our findings support previous observations showing management of patient with PE are more often treated in accordance with guidelines than patients who experienced DVT. 9 CAT is most often managed in the oncology departments, which also reflected in our results. CAT was for most patients managed and followed in the oncology department. However, follow-up was characterized as passive during active oncology treatment, and only one-third of the patients were followed up in a specialized thrombosis clinic. In another contemporary survey distributed among both cardiologists, oncologists and hematologists, only 21% of physicians felt confident in selecting an appropriate anticoagulant strategy for cancer patients. 16 This underlines an alarming lack of clinical ownership of the topic of cancer-associated thrombosis. Patients with cancer-associated thrombosis have also frequently reported that they feel ill-informed about their thrombotic event and the associated treatment. 28 Although cardio-oncology is still an emerging field, designated cancer-thrombosis services have been shown to improve consistency and safety of anticoagulant management of CAT. 32 33 A future multidisciplinary approach to CAT between oncologists, hematologists, surgeons, and cardiologists as well as increasing awareness on cancer thrombosis guidelines, likely has the potential to further improve treatment patterns and eliminate unnecessary knowledge deficits and uncertainties among both patients and physicians. 34

## Limitations

Some factors in the study design may potentially have influenced the reported management of CAT in Danish oncology departments: First, self-selection bias is evident as completion of the survey was open and voluntary, and physicians choosing to participate were likely to have a specific interest in CAT, which may have led to an overestimation of guideline knowledge. ﻿Second, reporting of the exact response rate was not applicable given the open recruitment strategies used, but the estimated response rate was 30% which is the average in email-surveys and considered acceptable. 13 Further, the survey did include respondents from all Danish regions and various levels of seniority. The study was underpowered to assess regional variation in physicians' knowledge and clinical treatment patterns. Third, the survey was distributed among oncologists, and cardiologists are often part of the decision-process in the management of CAT, which was also indicated in the free-text responses in the survey. Further, as this was the first study to describe the management of CAT in Danish oncology departments, we cannot evaluate whether introducing practice guidelines in Denmark has changed clinical management. Finally, the survey was distributed prior to the recent establishment of DOACs as an accepted treatment option for CAT thrombosis, which was not a recommended option in then applicable 2017 Danish guidelines. Of note the survey was not designed to elucidate perioperative thromboprophylaxis patterns, where treatment gaps are also evident. 35 Likewise, Danish guidelines actually allow for use of primary thromboprophylaxis for selected ambulatory cancer patients, but this survey did not cover this aspect, which recent data suggest is rarely used. 36

## Conclusion

Clinical practice guidelines offer evidence-based, best practice standards, but they are only effective if adopted throughout the health care system. Wide variability in the daily clinical management of CAT was demonstrated through this survey. Particularly, there is potential to increase awareness of available guidelines, standardized use of inpatient thromboprophylaxis, and organized treatment and follow-up in a multidisciplinary setting, which would potentially improve management of CAT in Denmark.
