# Supplementary material for: Management of Cancer-Associated Venous Thrombosis: A Nationwide Survey among Danish Oncologists
Source: TH Open. 2021 Jun 16;5(2):e188–94. doi: 10.1055/s-0041-1729754 (PMC8208837; doi:10.1055/s-0041-1729754)
Supplement: Supplementary file 1 — Supplementary Material [file 10-1055-s-0041-1729754-s200103.pdf]

Supplementary Table 1

Demographics

Geographical region of employment (*n* = 142)	% (*n*)
Capital region of Denmark	28.9 (41)
Central Denmark region	21.8 (31)
North Denmark region	9.9 (14)
Region of Southern Denmark	32.4 (46)
Region Zealand	7.0 (10)
Position (*n* = 142)	% (n)
Chief physician	36.6 (52)
Senior registrar	23.9 (34)
Registrar	25.4 (36)
Intern/resident	14.1 (20)
Oncology subspecialty[a] (*n* = 142)	% (*n*)
Breast cancer	18.3 (26)
Lung cancer	19.0 (27)
Gastrointestinal cancer	19.0 (27)
Head-neck cancer	8.5 (12)
Urological	9.2 (13)
Unspecified, shifting between subspecialities	19.7 (28)
Cancer of unknown primary, palliation, sarcoma, gynecological cancer, neuroendocrine tumor, central nervous system tumor, and malignant melanoma	<4

[a]The response format was a free-text box, allowing participants to give an open answer.

Treatment of venous thromboembolism in the oncology department

Are patients with DVT treated in the department? (*n* = 142)	% (*n*)
Yes	98.6 (140)
No	1.4 (2)
Are patients with pulmonary embolism treated in the department? (*n* = 142)	% (*n*)
Yes	62.7 (89)
Yes, but only if the patient is hemodynamically stable	35.9 (51)
No	1.4 (2)
Patients with pulmonary embolism who are not hemodynamically stable are treated in the: (*n* = 51)	% (*n*)
Medical department	0
Acute medical unit	9.8 (5)
Cardiology department	86.3(44)
Other (specify)	3.9 (2)

Diagnosis

What is the most commonly used strategy to assess the clinical probability of DVT? (*n* = 140) (Danish guidelines do not provide suggestions on diagnostic tools)	% (*n*)
Wells' score	15.0 (21)
Other scoring system	0
Clinical assessment (no scoring system)	80.7 (113)
Don't know	4.3 (6)
What is the most commonly used strategy to assess the clinical probability of pulmonary embolism? (*n* = 140) (Danish guidelines do not provide suggestions on diagnostic tools)	% (*n*)
Wells' score	5.0 (7)
Geneva score	0
Other scoring system	0
Clinical assessment (no scoring system)	94.3 (132)
Don't know	0.7 (1)

Anticoagulant treatment

First-line treatment in the department of patients with active cancer and cancer associated venous thrombosis: (*n* = 140) (Guidelines recommend weight-based LMWH)	% (*n*)
Vitamin K antagonist (warfarin)	0
Low-molecular-weight heparin (weight-based)	98.6 (138)
NOAC (Xarelto, Lixiana, Pradaxa, Eliquis)	0.7 (1)
Don't know	0.7 (1)
Second-line treatment in the department of patients with active cancer and cancer-associated venous thrombosis: (*n* = 140) (Guidelines recommended VKA at the time)	% (*n*)
Vitamin K-antagonist (warfarin)	12.1 (17)
Low-molecular-weight heparin (weight-based)	6.4 (9)
NOAC (Xarelto, Lixiana, Pradaxa, Eliquis)	36.4 (51)
Don't know	45.0 (63)
Specification of NOAC (second-line treatment): (*n* = 36)	% (*n*)
Xarelto	72.2 (26)
Lixiana	2.8 (1)
Pradaxa	11.1 (4)
Eliquis	13.9 (5)

Abbreviations: DVT, venous thromboembolism; LMWH, low-molecular-weight heparin; NOAC, novel oral anticoagulants; VKA, vitamin K antagonists.

 © 2021. The Author(s).

Treatment duration

Patients with active cancer and new-onset DVT are by default treated: (chose answer based on the common practice in the department; $n = 139$)<br>(Guidelines recommend continued treatment in patients with active cancer. Treatment recommendations are irrespective of type of VTE (DVT/PE)]	% (*n*)
3 months	3.6 (5)
6 months	36.0 (50)
12 months	2.2 (3)
As long as the patient have active cancer	58.3 (81)
Other, specify:	0
Patients with active cancer and new-onset pulmonary embolism are by default treated: (chose answer based on the common practice in the department) ($n = 139$)<br>(Guidelines recommend continued treatment in patients with active cancer)	% (*n*)
3 months	0
6 months	20.1 (28)
12 months	2.2 (3)
As long as the patient have active cancer	77.7 (108)
Other, specify:	0

Abbreviations: DVT, venous thromboembolism; PE, pulmonary embolism; VTE, venous thromboembolism.

Increased bleeding risk

Our treatment choice in the department for patients with increased bleeding risk is: ($n = 139$)<br>(Guidelines recommend individual assessment)	% (n)
Vitamin K-antagonist (warfarin)	0
Low-molecular-weight heparin (weight-based)	40.3 (56)
NOAC (Xarelto, Lixiana, Pradaxa, Eliquis)	0
Individual assessment in terms of type and intensity of anticoagulant treatment	49.6 (69)
Don't know	10.1 (14)

Abbreviation: NOAC, novel oral anticoagulant.

Recurrence while in anticoagulant treatment

Our treatment choice in the department if patients have a recurrent VTE while treated with LMWH: ($n = 138$)<br>[Guidelines recommend dose escalation of LMWH]	% (n)
Increases dosage of LMWH	70.3 (97)
Switches to Vitamin K antagonist (warfarin)	0.7 (1)
Switches to NOAC (Xarelto, Lixiana, Pradaxa, Eliquis)	2.2 (3)
Other, specify:<br>• Confers with center for thrombosis and hemostasis<br>• Always confers with the thrombosis clinic/cardiologist<br>• Askes the cardiologists/thrombosis center, who have specialized knowledge.<br>• The hemostasis specialist on guard is contacted for advice.<br>• Confer with center of thrombosis and hemostasis<br>• Fortsæt med gængse dosering LMH efter vægt i terapeutisk dosering (continue LMWH without dose-adjustment)<br>• Increase dosage to 125%<br>• Increase dosage<br>• Referral to the specialized department to determine cause. Additionally, switches to NOAC<br>• Dividing current dose into two<br>• Continuing current treatment<br>• Treatment twice daily with LMWH<br>• Seeks specialist advice<br>• Dividing LMWH in two daily dosage, increase dosage of LMWH, and contacting the center of thrombosis and hemostasis<br>• Dose is divided into two	10.9 (15)
Don't know	15.9 (22)

Abbreviations: DVT, venous thromboembolism; LMWH, low-molecular-weight heparin; NOAC, novel oral anticoagulants; VTE, venous thromboembolism.

Follow-Up

Follow-up after cancer associated thrombosis ($n = 137$)<br>(Not specified in the Danish guidelines)	% (*n*)
The patients are seen in the outpatient clinic at the oncology department for follow-up/control	44.5 (61)
The patient is referred to follow-up/control in a specialized thrombosis clinic/unit	34.3 (47)
Follow-up/control is handled by the general practitioner	13.9 (19)
Don't know	7.3 (10)

 © 2021. The Author(s).

Thromboprophylaxis

The question regards all patients in the department (including non-CT patients)<br>All patients with active cancer, who are hospitalized and immobilized receives by default thromboprophylaxis (unless it is contraindicated)? ($n = 139$)<br>(Guidelines recommend prophylaxis unless contraindicated)	% (n)
Yes	27.3 (38)
Yes, if the patient has evident risk factors for VTE (e.g., prior DVT or PE)	28.1 (39)
Only exceptionally	44.6 (62)

Abbreviations: CT, cancer-associated venous thrombosis; DVT, venous thromboembolism; PE, pulmonary embolism; VTE, venous thromboembolism.

Cancer-associated venous thrombosis guideline

Does the department have a clinical practice guideline for cancer-associated venous thrombosis? ($n = 142$)	% (n)
Yes	52.1 (74)
No	11.3 (16)
Don’t know	36.6 (52)

 © 2021. The Author(s).
